# Socioeconomic factors affecting breast and cervical cancer screening compliance in Asian National Cancer Centers Alliance countries: a systematic review

**DOI:** 10.4178/epih.e2025050

**Published:** 2025-08-28

**Authors:** Seowoo Bae, Ye Ji Kang, Jeonghoon Ahn, Bo-Hyoung Jang, Kui Son Choi, Hyeon Ji Lee, Mina Suh

**Affiliations:** 1 National Cancer Control Institute, National Cancer Center, Goyang, Korea; 2 Department of Health Convergence, Ewha Womans University, Seoul, Korea; 3 Department of Preventive Medicine, Kyung Hee University College of Korean Medicine, Seoul, Korea; 4 Graduate School of Cancer Science and Policy, National Cancer Center, Goyang, Korea

**Keywords:** Early detection of cancer, Breast neoplasm, Uterine cervical neoplasm, Socioeconomic factors, Systematic review

## Abstract

Breast and cervical cancers are the most frequently diagnosed cancers in women. The Asian National Cancer Centers Alliance (ANCCA) has strengthened cancer control efforts in the Asia region; however, only a few countries have achieved sufficient participation rates. This systematic review aimed to synthesize the existing evidence on socioeconomic factors influencing women’s compliance with breast and cervical cancer screening in ANCCA countries. This study was conducted as a systematic review, with studies collected from PubMed, Cochrane Library, Scopus, and Embase. All included studies employed cross-sectional designs to identify socioeconomic factors affecting compliance with breast or cervical cancer screening. Study selection, quality assessment, and data extraction were carried out by 2 independent reviewers with cross-checking. In total, 48 studies were reviewed. Education level and family history were associated with participation in breast cancer screening, while education level, household income, marital status, and medical insurance were linked with cervical cancer screening. When stratified by Human Development Index (HDI) level or by the presence of a National Cancer Screening Program, differences were observed in the factors influencing screening compliance. Nevertheless, higher education consistently correlated with higher screening rates for both cancer types, regardless of HDI level. This systematic review identified multiple socioeconomic factors that shape breast and cervical cancer screening compliance in Asian countries. To reduce disparities in participation, tailored multi-strategy approaches adapted to each country’s specific context are required. These findings may provide useful evidence for future research and policy initiatives aimed at addressing health equity issues.

## GRAPHICAL ABSTRACT

[Fig f3-epih-47-e2025050]

## Key Message

Breast and cervical cancer screening compliance among women is influenced by various socioeconomic factors. Higher education consistently increased screening participation for both cancers, whereas other factors differed by cancer type. To reduce disparities and promote health equity, multi-strategy approaches tailored to each country’s specific context are required.

## INTRODUCTION

Breast and cervical cancers are the most commonly diagnosed cancers among women worldwide [1], contributing disproportionately to morbidity and mortality in low- and middle-income countries (LMICs) [2]. In 2022, breast cancer accounted for approximately 2.3 million new cases, representing 11.6% of all cancers, and around 666,000 deaths (6.9%). Cervical cancer contributed 661,021 new cases (3.3%) and 348,189 deaths (3.6%) [1].

To address this global health burden, the World Health Organization (WHO) recommends organized national screening programs to ensure early detection and timely treatment of breast and cervical cancers [2,3]. Specifically for cervical cancer, the WHO has set a goal of elimination by 2030, emphasizing widespread human papillomavirus (HPV) vaccination alongside regular screening [2].

With nearly 60% of the world’s population residing in Asia, the region carries a considerable cancer burden, underscoring the urgent need for effective control strategies [4]. The Asian National Cancer Centers Alliance (ANCCA) has been pivotal in fostering regional collaboration, disseminating best practices, and supporting evidence-based policy implementation across member countries to improve cancer control, including screening for breast and cervical cancers [5,6].

Despite these efforts, prevention and management strategies remain highly variable across countries [5,6]. ANCCA’s comparative analysis found that although 15 member countries (71%) provide breast cancer screening, only 8 (38%) operate organized programs, often substituting clinical breast examinations (CBE) or ultrasound for mammography due to infrastructure constraints [5]. Breast cancer screening rates vary widely, ranging from 6.7% in Bangladesh to 64% in Korea [6]. For cervical cancer, most countries offer national screening programs, yet participation remains low, with only 5 reporting coverage above 50% [5].

Disparities in screening compliance arise not only from healthcare infrastructure but also from broader socioeconomic and structural determinants, including income, education, health literacy, policy priorities, and accessibility of care. Socioeconomic factors are well established as major influences on participation. For instance, individuals with higher levels of education are more likely to undergo screening. Although several studies have examined these issues within individual countries or regions, comparative research that integrates findings across Asia remains limited [7].

Therefore, this study aimed to analyze the socioeconomic factors influencing participation in breast and cervical cancer screening across ANCCA member countries, while also comparing differences in the impact of these factors between cancer types. In addition, countries were grouped by income level and by the presence of a national screening program to examine patterns among those with similar healthcare infrastructure.

## MATERIALS AND METHODS

The Preferred Reporting Items for Systematic reviews and Meta-Analysis (PRISMA) statement was used as a guideline in conducting this study. The study protocol was registered in PROSPERO on May 3, 2024 (ID No. CRD42024544549).

The purpose of this review was to identify socioeconomic factors associated with compliance with breast or cervical cancer screening in ANCCA member countries, focusing on women aged 20 years and older. Guided by the conceptual frameworks of the World Report on Social Determinants of Health Equity [8] and the Health at a Glance 2023 report [9], this study specifically selected socioeconomic variables including age, household income, education level, employment status, residential area (urban vs. rural), marital status, family history of cancer, and health insurance coverage as potential determinants of screening behavior. These factors represent internationally recognized social determinants of health, which shape healthcare access, participation in preventive services, and uptake of cancer screening.

Because of the heterogeneity in study designs and outcome measures among the included studies, a qualitative synthesis was employed. A socioeconomic factor was considered associated with screening compliance if more than half of the studies that examined it reported a statistically significant association. This threshold was established to ensure consistency in interpretation across diverse study contexts. The primary outcome was screening compliance for breast or cervical cancer, and only cross-sectional studies were included. Although the initial search did not restrict study design, most eligible studies identified during screening were cross-sectional. As the goal of this review was to assess screening compliance at a single point in time, cross-sectional studies were deemed most appropriate. To maintain methodological consistency and minimize heterogeneity, only cross-sectional studies were ultimately retained.

### Search strategy

PubMed, Cochrane Library, Embase, and Scopus were searched on March 22, 2024, using the following strategy:

(Breast neoplasms[MESH] OR “breast cancer” OR Uterine cervical neoplasms[MESH] OR “cervical cancer”) AND (Early detection of cancer[MESH] OR screen* OR Mammography[MESH] OR pap smear OR “pap test”) AND (“screening adherence” OR “compliance adherence” OR “participation” OR “adherence” OR “compliance”) AND (“factor” OR “facilitator” OR “barrier” OR “socioeconomic” OR “inequality”) AND (“Afghanistan” OR “Armenia” OR “Azerbaijan” OR “Bahrain” OR “Bangladesh” OR “Bhutan” OR “Brunei” OR “Cambodia” OR “China” OR “Cyprus” OR “Georgia” OR “India” OR “Indonesia” OR “Iran” OR “Iraq” OR “Israel” OR “Japan” OR “Jordan” OR “Kazakhstan” OR “Kuwait” OR “Kyrgyzstan” OR “Laos” OR “Lebanon” OR “Malaysia” OR “Maldives” OR “Mongolia” OR “Myanmar” OR “Nepal” OR “Korea” OR “Oman” OR “Pakistan” OR “Palestine” OR “Philippines” OR “Qatar” OR “Russia” OR “Saudi Arabia” OR “Singapore” OR “Sri Lanka” OR “Syria” OR “Taiwan” OR “Tajikistan” OR “Thailand” OR “Timor-Leste” OR “Turkey” OR “Turkmenistan” OR “United Arab Emirates” OR “Uzbekistan” OR “Vietnam” OR “Yemen”). The search strategy was designed to incorporate the target cancer, screening outcomes, socioeconomic determinants, and the full list of Asian countries.

Studies investigating participation in breast or cervical cancer screening were included, and only those published after 2010 were considered. Studies were excluded if: (1) the study population was not the general population (e.g. high-risk groups with adenomas or a history of cancer, or healthcare providers); (2) they were unrelated to breast or cervical cancer; (3) they lacked outcomes related to screening adherence or compliance; (4) they did not include comparisons across levels of socioeconomic factors (e.g., age, income level, education level, employment status, residential area [urban or rural], marital status, health insurance coverage, or family history); (5) they did not have a cross-sectional design; (6) they did not involve human participants (e.g., animal studies); (7) they were not conducted in 1 of the 21 ANCCA countries; (8) they were not written in English; (9) they were in the gray literature (e.g., research reports, conference materials, or unpublished manuscripts); (10) they were study protocols; (11) the full text was not available; and (12) there were other reasonable grounds for exclusion (e.g. conference abstracts).

Titles and abstracts were screened in the first round, followed by full-text review. Both rounds were independently conducted by 2 researchers. Disagreements were resolved by re-evaluation or consultation with a third researcher until consensus was reached.

### Quality assessment

The quality of the included studies was assessed using the Appraisal Tool to Assess the Quality of Cross-Sectional Studies developed by the *British Medical Journal*. This tool, created through a Delphi process, includes 20 items. A scoring system was applied, assigning 1 point for “yes” and 0 points for both “no” and “cannot tell/do not know,” resulting in a maximum score of 20. Studies scoring 19-20 points were classified as very low risk of bias, 17-18 as low risk, 15-16 as moderate risk, and below 14 as high risk of bias. In this review, studies scoring below 16 were excluded.

### Systematic review

A qualitative synthesis was performed to determine whether each socioeconomic factor had a significant effect on participation in breast or cervical cancer screening. For factors found to significantly influence screening participation, results were summarized using odds ratios (ORs) with 95% confidence intervals (CIs).

### Subgroup analysis

Countries were categorized by their economic and social development levels using the Human Development Index (HDI), developed by the United Nations Development Programme. The HDI is a composite index integrating life expectancy, education (mean and expected years of schooling), and income (gross national income per capita). Countries are classified into 4 categories, from group 1 (very high) to group 4 (low). In this study, subgroup analyses compared group 1 (very high) with groups 2-3 (high and medium). None of the included countries fell into group 4 (low).

In addition, a subgroup analysis was conducted for breast cancer based on whether countries provided a National Cancer Screening Program (NCSP). Studies conducted prior to the implementation of such programs were excluded from this analysis. For cervical cancer, subgroup analysis was not performed because all included studies were from countries that had already implemented national screening programs.

### Ethics statement

No ethical approval or informed consent were not required since this study was based on published articles only, and did not involve any human subject data.

## RESULTS

### Search outcomes

**Figure 1.**
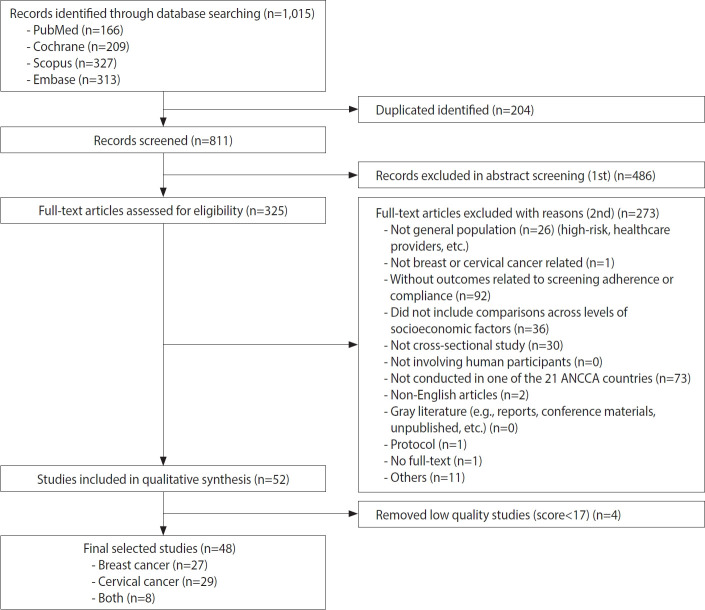
Flow chart of the study selection process. ANCCA, Asian National Cancer Centers Alliance.

A total of 1,015 articles were identified (PubMed: 166, Cochrane: 209, Scopus: 327, and Embase: 313), with 204 duplicates removed. After title and abstract screening, many studies were excluded, leaving 325 articles for full-text review. Of these, 52 studies met the inclusion criteria. Following quality assessment, 4 studies were excluded for failing to meet the predefined threshold of 16 points. Consequently, 48 studies were included in the final analysis. The process of study selection is illustrated in Figure 1.

### Study characteristics

All 48 studies used a cross-sectional design to evaluate socioeconomic factors associated with participation in breast or cervical cancer screening. Among these, 27 studies examined breast cancer screening [10-36], while 29 studies focused on cervical cancer screening [11,13,15,18,19,27,29,31,37-57]. Eight studies investigated both breast and cervical cancer screening and were classified under both categories. In total, 12 countries were represented in this review.

Table 1 summarizes the characteristics of studies on breast cancer screening. Korea and China contributed the highest number of studies, with 6 each. Iran contributed 5 studies, while Indonesia, Thailand, Japan, Mongolia, and Malaysia provided between 1 and 3 studies each. Screening methods varied across studies, including breast self-examination, CBE, mammography, and ultrasonography. Sample sizes ranged from 206 to 4,042,332 participants.

Among the 29 studies on cervical cancer screening, China and Korea contributed the most, with 8 and 5 studies, respectively. Iran contributed 4 studies, Malaysia 3, and Thailand 2, while several other countries contributed 1 study each. Screening modalities included Pap smear testing, pelvic examination, and HPV testing. Sample sizes ranged from 77 to 21,422 participants (Table 2).

### Age associations with participation in breast and cervical cancer screening

Many studies on breast cancer focused on women aged 40 years and older. Of the 23 studies that analyzed age as a factor influencing screening participation, 17 demonstrated significant associations. Overall, screening rates tended to be lower in age groups younger than 40 or older than 60, with the highest rates typically among women in their 40s and 50s. For instance, Gang et al. [10] reported that women aged 40-49 had higher mammography screening rates than those under 39 (OR, 2.37; 95% CI, 1.10 to 5.12). Conversely, Teo et al. [28] found that women aged 50 and older were less likely to undergo screening compared with those younger than 50 (OR, 0.57; 95% CI, 0.34 to 0.94).

For cervical cancer, study populations were generally younger than those in breast cancer studies. Among the 23 studies that analyzed age, 14 reported significant associations, but no consistent directional trend was observed across age groups. The influence of age on screening compliance for each study is detailed in Supplementary Materials 1 and 2.

### Socioeconomic factors associated with participation in breast and cervical cancer screening

Table 3 presents the socioeconomic factors associated with participation in breast and cervical cancer screening as identified in the included studies.

For breast cancer screening, educational level and family history of breast cancer were most frequently identified as significant determinants. More than half of the studies found that women with higher education levels or with a family history of breast cancer were more likely to participate in screening. For example, Anwar et al. [18] reported that women with at least a high school education were significantly more likely to undergo breast cancer screening compared to those with less education (OR, 4.26; 95% CI, 3.39 to 5.36) (Supplementary Material 3). Similarly, studies comparing women with no formal education to those with some education consistently found higher participation rates in the latter group [16,17,23,32,36] (Supplementary Material 3). With regard to family history, Grosse Frie et al. [16] found that women with a family history of breast cancer were more likely to participate in screening than those without (OR, 1.24; 95% CI, 1.10 to 1.39).

For cervical cancer, education level was again a major determinant, but additional factors were consistently associated with screening participation, including household income, marital status, and medical insurance coverage. For example, Lee et al. [11] reported that women in higher income quintiles were more likely to undergo cervical cancer screening than those in the lowest quintile, with adjusted ORs of 2.16 (95% CI, 1.08 to 4.31) for the fourth quintile and 3.39 (95% CI, 1.66 to 6.92) for the fifth quintile (Supplementary Material 4).

Marital status was also strongly associated with participation. Amin et al. [44] demonstrated that married women had substantially higher odds of screening compared with single women (OR, 45.84; 95% CI, 29.46 to 71.34) (Supplementary Material 5).

Furthermore, 4 studies consistently showed that women with medical insurance were more likely to participate in screening than those without coverage [18,40,45,48] (Supplementary Material 5).

### Socioeconomic factors affecting cancer screening participation rate by Human Development Index classification

**Figure 2.**
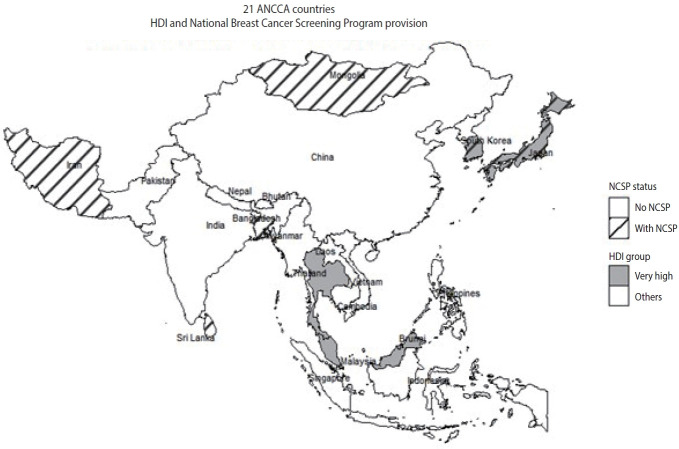
21 Asian National Cancer Centers Alliance (ANCCA) countries classified into Human Development Index (HDI) level and National Breast Cancer Screening Program provision. NCSP, National Cancer Screening Program.

As shown in Table 4, this study examined the socioeconomic factors influencing screening participation, stratified by the HDI group of each country. The classification of ANCCA member countries by HDI level is illustrated in Figure 2.

#### Breast cancer screening

In countries with very high HDI (group 1), including Singapore, Korea, Thailand, and Japan, education level and household income emerged as significant factors. In contrast, in countries with high to low HDI (groups 2-4), such as Iran, Indonesia, China, India, Mongolia, and Bhutan, screening participation was more strongly influenced by education level, employment status, and family history.

#### Cervical cancer screening

For cervical cancer screening, education level was the primary determinant in group 1 countries. In groups 2-4, a broader range of factors influenced participation, including education level, household income, employment status, and marital status.

The ORs for each factor with significant effects are presented in Supplementary Materials 6-10.

### Socioeconomic factors affecting breast cancer screening participation rate by National Cancer Screening Program implementation classification

ANCCA member countries were also categorized according to whether or not they had implemented a NCSP. The list of countries providing NCSP is shown in Figure 2.

Among countries with NCSP, household income was identified as a significant factor influencing screening participation, particularly in Singapore, Korea, and Japan. By contrast, in countries without NCSP, education level, employment status, and region of residence were consistently significant determinants. Specifically, education was identified as a significant factor in Indonesia, India, China, and Thailand; employment status in India and China; and region of residence in Indonesia and China (Table 4). The ORs for each significant factor are provided in Supplementary Materials 11 and 12.

## DISCUSSION

To our knowledge, this study is the only systematic review that explores socioeconomic factors influencing compliance with breast and cervical cancer screening across ANCCA countries. Unlike many studies that examine each cancer type separately, this research is notable in that it compares both cancers, highlighting how study designs vary and how the determinants of screening participation differ.

Before addressing the main findings, it is important to interpret the results with caution. Considerable heterogeneity existed across studies, including differences in screening methods and national contexts. As a result, only a qualitative synthesis was performed rather than a meta-analysis. A factor was considered to significantly influence screening compliance if more than half of the included studies reported such an effect. However, because these results are not based on pooled statistical estimates, they should not be regarded as conclusive evidence. Instead, they should be understood as indicative of potential trends.

In summary, education level and family history emerged as significant determinants of breast cancer screening compliance. For cervical cancer, education level was also a key factor, but additional determinants included household income, marital status, and medical insurance. These findings are consistent with evidence from the Korean National Cancer Screening Survey (2005-2015), which demonstrated greater socioeconomic inequalities in cervical cancer screening than in breast cancer screening [7]. Specifically, disparities in cervical cancer screening were quantified by a pooled slope index of inequality (SII) of 10.6% (95% CI, 8.1 to 13.2) and a relative index of inequality (RII) of 1.4 (95% CI, 1.3 to 1.6). By comparison, income-related disparities in breast cancer screening increased gradually over time, with a pooled SII of 5.9% (95% CI, 2.9 to 9.0) and an RII of 1.2 (95% CI, 0.9 to 1.3). These differences may partly reflect target age groups, as cervical cancer screening typically focuses on women in their 30s, who often exhibit greater socioeconomic variability, while breast cancer screening mainly targets women aged 40 and above, a group that generally has more stable socioeconomic profiles. Women in their 30s often have lower income levels compared with older age groups. Moreover, the higher proportion of single women in lower-income categories, who are less likely to participate in screening, may also contribute to these disparities [7].

When considering individual factors, education level consistently influenced screening compliance for both cancers. Notably, its effect was significant in both high-HDI and low-HDI countries. Education is a critical determinant of health literacy, which encompasses the ability to access, understand, and use health information and services for decision-making. Women with lower educational attainment face greater barriers to healthcare utilization, including cost, time, and distance. Consequently, higher levels of education are strongly associated with greater participation in cancer screening [58]. For example, in Korea, breast cancer screening showed the largest disparity by education level: among women aged over 40, the screening rate was 69.5% among those with education beyond college compared to 56.3% among those with only elementary schooling—a difference of 13.2 percentage points [59]. Similarly, participation in general health check-ups was higher among individuals with a college education compared with those with only elementary schooling (adjusted OR, 1.18) [60]. To reduce these disparities, countries should design screening strategies that address populations with lower educational attainment. This requires not only promotion and outreach initiatives but also multidimensional, tailored approaches that reflect the structure and characteristics of each healthcare system.

In breast cancer, but not in cervical cancer, family history was an important determinant of screening compliance. This difference likely reflects underlying etiology. Cervical cancer is primarily caused by infection with HPV, rather than genetic inheritance, whereas breast cancer has a well-documented hereditary component. A *Lancet* study reported that more than 12% of breast cancer patients had a family history of the disease [61]. *BRCA1* and *BRCA2* mutations are well-established genetic risk factors. Shih et al. [62] demonstrated that 42.9% of patients with multiple primary breast cancers carried either a *BRCA1* or *BRCA2* mutation (p<0.001). Compared with individuals without a family history, those with at least 1 first-degree relative with breast cancer had a significantly increased risk of developing the disease (hazard ratio [HR], 1.77; 95% CI, 1.58 to 1.97; p<0.001), and the risk more than doubled among those with 2 or more affected relatives (HR, 2.52; 95% CI, 1.83 to 3.47; p<0.001) [63].

In lower-HDI countries, employment status was a significant factor influencing screening compliance for both cancer types. Several explanations may account for why employed individuals are more likely to undergo cancer screening. These include access to workplace health screening programs, financial support for health check-ups, and greater awareness of the importance of screening through interactions with colleagues. Supporting evidence comes from a systematic review that found workplace-based health screening programs to be effective in improving knowledge and increasing screening uptake [64]. Similarly, a Korean study reported higher gastric cancer screening participation among full-time employees compared with non-regular workers or the self-employed. This was attributed to greater job security and more favorable working conditions among regular employees, which facilitated access to health screenings [65].

In countries offering NCSPs, household income remained a significant factor, whereas it was not in countries without such programs. Initially, it was anticipated that financial support through national programs would reduce disparities in screening access by income. However, the findings indicate that household income continues to exert an influence even in countries with national programs. This may be explained by persistent inequalities in the availability of private screening services. Indeed, participation in national screenings has been reported to be higher among lower-income groups, while higher-income individuals more frequently use private screening options [66]. Another explanation is that most of the countries with national screening programs in this study—Korea, Japan, and Singapore—belonged to HDI group 1. In this group, household income was consistently associated with screening compliance, suggesting that the inclusion of many high-HDI countries implementing NCSP contributed to the observed results.

This study has several limitations. First, the included studies presented quality concerns, as all were cross-sectional in design, which is inherently lower in strength of evidence compared with randomized controlled trials. Moreover, differences in study settings (e.g., target age groups and screening methods) and variations in reporting require caution in interpreting the synthesized findings. For breast cancer in particular, screening rates vary depending on the method used, and the factors influencing participation may therefore differ. This issue warrants further investigation in follow-up studies. Second, although the search encompassed 21 ANCCA countries, only 12 countries (10 for breast cancer screening and 12 for cervical cancer screening) were represented in the final analysis. Nonetheless, these countries spanned a broad range of HDI levels and healthcare systems, lending relevance to this study’s exploration of socioeconomic factors across the Asian region. Third, subgroup analyses were limited by small numbers of studies for certain variables. For example, within the HDI group 1 subgroup for breast cancer, only 1 or 2 studies examined factors such as region of residence and family history, making it difficult to determine their significance with confidence. Finally, this review was restricted to socioeconomic variables and did not include other potentially important determinants. Screening compliance is also shaped by knowledge, attitudes, healthcare environments, and system-level characteristics. For instance, in countries such as India and Malaysia, screening programs are either unavailable at the national level or limited to specific cancers, and there is often no consistent system for follow-up diagnosis and treatment after abnormal findings. These limitations contribute to low participation rates and large regional disparities [6]. Future research should therefore incorporate a wider range of variables, including healthcare system factors, to enable a more comprehensive analysis.

## CONCLUSION

This systematic review identified socioeconomic factors influencing compliance with breast and cervical cancer screening in 12 Asian countries, based on evidence synthesized from multiple quantitative studies. For breast cancer, education level and family history were significant determinants of screening participation, while for cervical cancer, education, income level, marital status, and health insurance coverage were influential factors. However, the specific factors varied depending on HDI classification and the presence of a NCSP. To reduce disparities in screening compliance, multi-strategy approaches tailored to each country’s unique context are required. Furthermore, future studies should incorporate a broader set of determinants—including knowledge, attitudes, and healthcare system characteristics—to strengthen efforts promoting breast and cervical cancer screening across the Asian region.

## Figures and Tables

**Figure f3-epih-47-e2025050:**
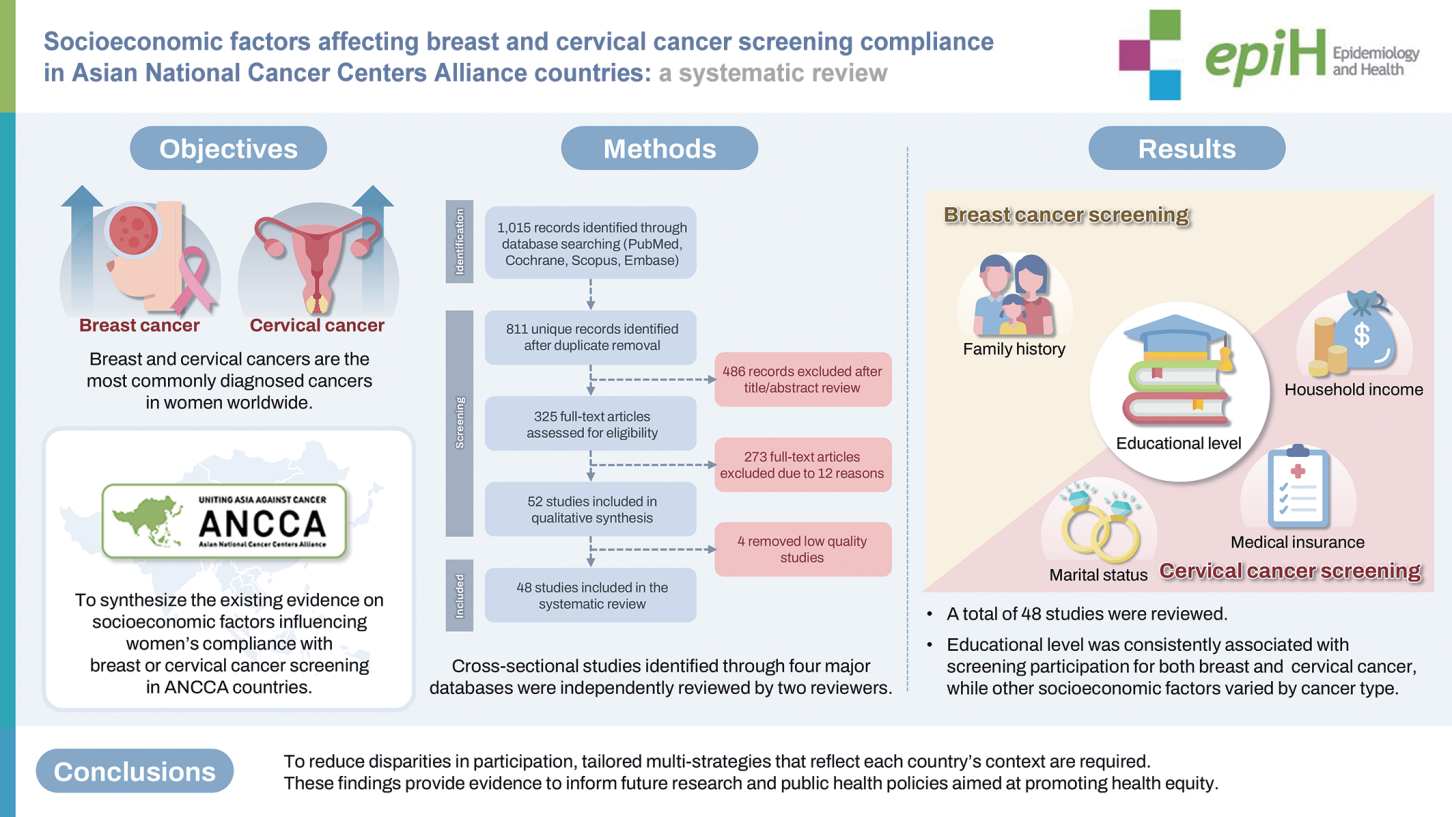


**Table 1.** Summary of the 27 included studies about breast cancer screening participation among women in Asia

Study	Country	Study design	Sample size (n)	Age of participants (yr)	Study period	Screening method (BSE | CBE | Mammography)	Quality score
Gang et al., 2013 [10]	China	Cross-sectional	406	≥20	2011	Mammography	19
Lee et al., 2015 [11]	China	Cross-sectional	5,735	50-74	2007-2010	Mammography	17
Leung et al., 2012 [12]	China	Cross-sectional	1,533	≥60	2006	Mammography, CBE, BSE	19
Sun et al., 2022 [13]	China	Cross-sectional	3,500	18-64	2018	Mammography, CBE	18
Wang et al., 2013 [14]	China	Cross-sectional	53,513	≥18	2010	Any method	20
You et al., 2019 [15]	China	Cross-sectional	6,520	36-65	2013	Mammography, CBE	18
Grosse Frie et al., 2013 [16]	India	Randomized controlled trial, cross-sectional	52,011	30-69	2006	BSE	18
Kulkarni et al., 2019 [17]	India	Cross-sectional	14,770	30-64	2010-2014	CBE	19
Anwar et al., 2018 [18]	Indonesia	Cross-sectional	5,397	≥40	2014-2015	BSE	18
Ahmadipour et al., 2016 [19]	Iran	Cross-sectional	240	18-64	2015	BSE, CBE, mammography	17
Allahverdipour et al., 2011 [20]	Iran	Cross-sectional	414	≥40	2007	Mammography	18
Aminisani et al., 2016 [21]	Iran	Cross-sectional	561	≥40	2014	Mammography	19
Ghanbari et al., 2020 [22]	Iran	Cross-sectional	1,472	15-45	2017	BSE, CBE, mammography	17
Samah et al., 2012 [23]	Iran	Cross-sectional	400	35-69	2009	Mammography	17
Okui, 2021 [24]	Japan	Cross-sectional	6,031	40-69	2010, 2013	Mammography	18
			2010: 2,817				
			2013: 3,214				
Tsunematsu et al., 2013 [25]	Japan	Cross-sectional	3,200	20-69	2012	Mammography	18
Yusof et al., 2014 [26]	Malaysia	Cross-sectional	206	40-74	2011	Mammography	18
Yerramilli et al., 2015 [27]	Mongolia	Cross-sectional	1,193	≥30	2010	BSE	19
Teo et al., 2013 [28]	Singapore	Cross-sectional	208	40-75	2011	Mammography	18
Wee et al., 2012 [29]	Singapore	Cross-sectional	1,383	≥40	2009-2011	Mammography	18
Hahm et al., 2011 [30]	Korea	Cross-sectional	777	≥40	2007	Mammography or ultrasonography	17
Lee et al., 2010 [31]	Korea	Cross-sectional	2,609	≥40	2005	Mammography or ultrasonography	17
Lee et al., 2010 [32]	Korea	Cross-sectional	2,583	≥40	2005	Mammography or ultrasonography	18
Nari et al., 2023 [33]	Korea	Cross-sectional	4,042,332	≥40	2018	Mammography	17
Oh et al., 2011 [34]	Korea	Cross-sectional	2,511,976	≥40	2005-2008	Mammography	19
Son et al., 2017 [35]	Korea	Cross-sectional	1,193	≥40	2012	Mammography	20
Mukem et al., 2014 [36]	Thailand	Cross-sectional	18,474	≥20	2007	BSE, CBE, mammography	17

BSE, breast self-exam; CBE, clinical breast exam.

**Table 2.** Summary of the 29 included studies about cervical cancer screening participation among women in Asia

Study	Country	Study design	Sample size (n)	Age of participants	Study period	Screening method (Pap smear test | Pelvic examination | HPV test)	Quality score
Baussano et al., 2014 [37]	Bhutan	Cross-sectional	1,620	≥25	2011-2012	HPV test, Pap smear test	19
Gu et al., 2010 [38]	China	Cross-sectional	167	25-50	2007	Specific method N/R	17
Lee et al., 2015 [11]	China	Cross-sectional	5,823	25-69	2007-2010	Pelvic examination, Pap smear test, mammography	17
Lin et al., 2021 [39]	China	Cross-sectional	8,639	30-60	2015	HPV test	19
Lin et al., 2021 [40]	China	Cross-sectional	12,017	21-60	2011	Specific method N/R	20
			2011: 9,155		2014		
			2014: 2,862				
Liu et al., 2017 [41]	China	Cross-sectional	405	30-65	2015	Specific method N/R	17
Sun et al., 2022 [13]	China	Cross-sectional	3,500	18-64	2018	Specific method N/R	18
You et al., 2019 [15]	China	Cross-sectional	6,520	36-65	2013	Specific method N/R	18
Zhang et al., 2023 [42]	China	Cross-sectional	4,518	18-65	2022	Pap smear, HPV test, colposcopy	18
Anwar et al., 2018 [18]	Indonesia	Cross-sectional	1,058	≥40	2014-2015	Pap smear test	18
Kulkarni et al., 2022 [43]	India	Cross-sectional	21,422	30-65	2007-2012	Specific method N/R	17
Ahmadipour et al., 2016 [19]	Iran	Cross-sectional	240	18-64	2015	Pap smear test pelvic examination	17
Amin et al., 2020 [44]	Iran	Cross-sectional	15,975	≥18	2016	Pap smear test	20
Aminisani et al., 2016 [45]	Iran	Cross-sectional	561	≥40	2014	Pap smear test	17
Mosayebi et al., 2018 [46]	Iran	Cross-sectional	77	27-85	2014-2016	Pap smear test	20
Cui et al., 2022 [47]	Japan	Cross-sectional	816	20-39	2018	Any method	17
Al-Oseely et al., 2023 [48]	Malaysia	Cross-sectional	355	20-65	N/S	Pap smear test	17
Siraj et al., 2019 [49]	Malaysia	Cross-sectional	300	≥17	N/S	Pap smear test	18
Yunus et al., 2018 [50]	Malaysia	Cross-sectional	316	20-65	2013	Pap smear test	19
Yerramilli et al., 2015 [27]	Mongolia	Cross-sectional	1,193	≥30	2010	Pap smear test	19
Ranabhat et al., 2014 [51]	Nepal	Cross-sectional	607	≥18	2013	Pap smear test	18
Wee et al., 2012 [29]	Singapore	Cross-sectional	438	≥40	2009-2011	Pap smear test	18
Chang et al., 2017 [52]	Korea	Cross-sectional	3,734	15-39	2010-2012	Pap smear test	20
Chang et al., 2018 [53]	Korea	Cross-sectional	15,935	≥30	2014-2015	Pap smear test	18
Lee et al., 2010 [31]	Korea	Cross-sectional	3,413	≥30	2005	Pap smear test	17
Lee et al., 2013 [54]	Korea	Cross-sectional	17,105	≥30	1998–2010	Pap smear test	20
Shin, et al., 2022 [55]	Korea	Cross-sectional	3,925	20-39	2017-2020	Pap smear test	18
Visanuyothin et al., 2015 [56]	Thailand	Cross-sectional	595	30-60	2012	Pap smear test	17
Wongwatcharanukul et al., 2014 [57]	Thailand	Cross-sectional	547	30-60	2012	Pap smear test	18

N/R, not recorded; N/S, not specified; Pap, Papanicolaou; HPV, human papillomavirus

**Table 3.** Socioeconomic factors and associations (or non-associations) with participation in breast or cervical cancer screening

Socioeconomic factors	Breast cancer screening			Cervical cancer screening		
	Studies displaying a positive association (p<0.05)	Studies displaying a negative association (p<0.05)	Studies displaying no association (p≥0.05)	Studies displaying a positive association (p<0.05)	Studies displaying a negative association (p<0.05)	Studies displaying no association (p≥0.05)
Higher education^1,2^	[10-12,14-19,23,24,27,28,32,36]	-	[13,20-22,26,29,30,35]	[11,15,18,19,27,29,38-41,43-46,49,52,54,57]	-	[13,42,50,56]
Higher household income^2^	[11,15,24,28,29,30,35,36]	-	[13,16-19,23,26,31,32]	[11,15,18,19,31,39,40,42,52-54]	-	[13,29,41,48,50,56]
Being employed	[13,14,16,17,22-24]	-	[10,15,19,20,25,27,29,30,35]	[13,19,27,29,37,40,42,49,53]	[56]	[15,38,43,47,48,50,52,54]
Being married^2^	[13,16,17,24,25,36]	[12,14]	[11,15,18,20,21,23,26,30,32]	[13,37,38,40,42,44,47,55,56]	[49]	[11,15,18,39,41,48,50]
Urban residence	[11,18]	[27,30]	[14,20,32]	[11]	[53]	[18,42,44,51]
Medical insurance^2^	[18,22,23,32,36]	[20]	[11,13,26,34,35]	[18,40,45,48]		[11,13]
Family history^1^	[16,20,22,25]		[21,23,28]	[43]		[56]

1 Factors displaying positive association with breast cancer screening.

2 Factors displaying positive association with cervical cancer screening.

**Table 4.** Factors affecting cancer screening participation rate by HDI classification and NCSP provision classification

Classifications	Factors	Applicable countries	List of studies
HDI classification			
Breast cancer screening			
1 (very high)	Education level	Singapore, Korea, Thailand	[24,28,32,36]
	Household income	Singapore, Korea, Thailand, Japan	[24,28-30,35,36]
2-4 (high, moderate, low)	Education level	Iran, Indonesia, China, India, Mongolia	[10-12,14-19,23,27]
	Employment	India, Iran, China	[13,14,16,17,22,23]
	Family history	Iran, India	[16,20,22]
Cervical cancer screening			
1 (very high)	Education level	Korea, Malaysia, Singapore, Thailand	[29,49,52,54,57]
2-4 (high, moderate, low)	Education level	Iran, Indonesia, China, India, Mongolia	[11,15,18,19,27,38-41,43-46]
	Household income	Iran, Indonesia, China	[11,15,18,19,39,40,42]
	Employment	Iran, China, Mongolia, Bhutan	[13,19,27,37,40,42]
	Marriage status	Iran, China, Bhutan	[13,37,38,40,42,44]
NCSP provision classification			
Breast cancer screening			
National Program			
Provided	Household income	Singapore, Korea, Japan	[24,28-30,35]
Not provided	Education level	Indonesia, India, China, Thailand, China	[10-12,14-18,36]
	Employment	India, China,	[13,14,16,17]
	Region	Indonesia, China	[11,18]

HDI, Human Development Index; NCSP, National Cancer Screening Program.

## Supplementary materials

Supplementary Material 1. Age associations with participation in breast cancer screening

Supplementary Material 2. Age associations with participation in cervical cancer screening

Supplementary Material 3. Factors associated with participation in breast cancer screening (education level and family history)

Supplementary Material 4. Factors associated with participation in cervical cancer screening (education level, household income)

Supplementary Material 5. Factors associated with participation in cervical cancer screening (marriage status, medical insurance)

Supplementary Material 6. Socioeconomic factors associated with participation in breast cancer screening in HDI 1 group (Education level & Household income)

Supplementary Material 7. Socioeconomic factors associated with participation in breast cancer screening in HDI 2~3 group (Education level & Employment & Family history)

Supplementary Material 8. Socioeconomic factors associated with participation in cervical cancer screening in HDI 1 group (Education level)

Supplementary Material 9. Socioeconomic factors associated with participation in cervical cancer screening in HDI 2~3 group (Education level & Household income)

Supplementary Material 10. Socioeconomic factors associated with participation in cervical cancer screening in HDI 2~3 group (Marriage status & Employment)

Supplementary Material 11. Socioeconomic factors associated with participation in breast cancer screening in countries with National Screening Program (Household income)

Supplementary Material 12. Socioeconomic factors associated with participation in breast cancer screening in countries without National Screening Program (Education level & Employment & Region)
