# Supplementary material for: Socioeconomic factors affecting breast and cervical cancer screening compliance in Asian National Cancer Centers Alliance countries: a systematic review
Source: Epidemiol Health. 2025 Aug 28;47:e2025050. doi: 10.4178/epih.e2025050 (PMC12869128; doi:10.4178/epih.e2025050)
Supplement: Supplementary Material 1. — Age associations with participation in breast cancer screening [file epih-47-e2025050-Supplementary-1.docx]

**Supplementary Material 1. Age associations with participation in breast cancer screening**

| First author, publish year | Positive association tendency | Negative association tendency | Association in both sides |
| --- | --- | --- | --- |
| Allahverdipour, 2011 [20] | Above 40 (continuous) |  |  |
| Frie, 2013 [16] |  |  | 30-39<40-49  30-39>60-69 |
| Gang, 2013 [10] | Under 39<40-49 |  |  |
| Ghanbari, 2020 [22] | Under 30<30-35  Under 30<35-40  Under 30<above 40 |  |  |
| Hahm, 2010 [30] |  | Above 40 (Continuous) |  |
| Lee, 2015 [11] |  | 50-59>60-69  50-59>70-74 |  |
| Lee, 2010 [32] |  | 40-49>above 65 |  |
| Mukem, 2014 [36] |  |  | *BSE  20-24<40-44  25-29<40-44  40-44>55-59  40-44>above 60 |
|  |  |  | **CBE  20-24<40-44  25-29<40-44  30-34<40-44  40-44>above 60 |
|  |  |  | ***Mammography  40-44>above 60 |
| Nari, 2023 [33] |  |  | 40-49<50-59  40-49<60-69  40-49<70-79  40-49>above 80 |
| Oh, 2011 [34] |  |  | 40-49<50-59  40-49<60-69  40-49>above 70 |
| Okui, 2021 [24] |  | 40-49>60-69 |  |
| Samah, 2012 [23] |  |  | under 40<41-50  under 40>51-60  under 40>above 60 |
| Son, 2016 [35] | Above 40 (Continuous) |  |  |
| Sun, 2022 [13] | 18-34 < 35-64 |  |  |
| Teo, 2013 [28] |  | Under 50>above 50 |  |
| Wang, 2013 [14] |  | Under 30>above 70  30-39>above 70  40-49>above 70  50-59>above 70  60-69>above 70 |  |
| Yusof, 2014 [26] | 40-49<50-59  40-49<above 60 |  |  |
