# Supplementary material for: Socioeconomic factors affecting breast and cervical cancer screening compliance in Asian National Cancer Centers Alliance countries: a systematic review
Source: Epidemiol Health. 2025 Aug 28;47:e2025050. doi: 10.4178/epih.e2025050 (PMC12869128; doi:10.4178/epih.e2025050)
Supplement: Supplementary Material 2. — Age associations with participation in cervical cancer screening [file epih-47-e2025050-Supplementary-2.docx]

**Supplementary Material 2. Age associations with participation in cervical cancer screening**

| First author, publish year | Positive association tendency | Negative association tendency | Association in both sides |
| --- | --- | --- | --- |
| Al-Oseely, 2023 [48] | 20-34<35-49  20-34<50-65 |  |  |
| Aminisani, 2016 [45] |  | 40-49>above 60 |  |
| Baussano, 2014 [37] |  | 25-34>35-44 |  |
| Chang, 2017 [52] | 15-29<30-39 |  |  |
| Chang, 2018 [53] | Under 39<40-49  Under 39<above 50 |  |  |
| Cui, 2022 [47] |  | 25-29>35-39 |  |
| Kulkarni, 2022 [43] |  | 30-39>40-49  30-39>50-59  30-39>above 60 |  |
| Lee, 2015 [11] |  | 25-39>50-59  25-39>60-69 |  |
| Lin, 2021 [39] | 30-39<40-49 |  |  |
| Lin, 2021 [40] | 21-30<41-60 |  |  |
| Liu, 2017 [41] | 30-44<45-54  30-44<55-65 |  |  |
| Siraj, 2019 [49] |  | 17-40>51-60  17-40>above 60 |  |
| Sun, 2022 [13] | 18-34<35-64 |  |  |
| Zhang, 2023 [42] | 18-30<36-40  18-30<41-45  18-30<46-50  18-30<51-65 |  |  |
