# Supplementary material for: Socioeconomic factors affecting breast and cervical cancer screening compliance in Asian National Cancer Centers Alliance countries: a systematic review
Source: Epidemiol Health. 2025 Aug 28;47:e2025050. doi: 10.4178/epih.e2025050 (PMC12869128; doi:10.4178/epih.e2025050)
Supplement: Supplementary Material 3. — Factors associated with participation in breast cancer screening (education level and family history) [file epih-47-e2025050-Supplementary-3.docx]

**Supplementary Material 3.** **Factors associated with participation in breast cancer screening (education level and family history)**

|  | Education level | | Family history | |
| --- | --- | --- | --- | --- |
| First author, publish year | Group | OR (95% CI) | Group | OR (95% CI) |
| Ahmadipour, 2016 [19] | Below high school graduate (ref) vs others | 7.23 (1.22-42.98) |  |  |
| Allahverdipour, 2011 [20] |  |  | no(ref) vs yes | 5.58 (1.27-24.52) |
| Anwar, 2018 [18] | Below high school graduate (ref) vs others | 4.26 (3.39-5.36) |  |  |
| Frie, 2013 [16] | None (ref) vs primary  vs secondary  vs high school  vs university or higher | 1.76 (1.47-2.10)  2.62 (2.17-3.17)  4.62 (3.53-6.06)  7.86 (5.47-11.30) | no(ref) vs yes | 1.24 (1.10-1.39) |
| Gang, 2013 [10] | Highly educated (ref) vs low educated | 0.40 (0.21-0.77) |  |  |
| Ghanbari, 2020 [22] |  |  | no(ref) vs yes | 1.60 (1.19-2.19) |
| Kulkarni, 2019 [17] | None (ref) vs primary  vs secondary  vs high school | 1.41 (1.13-1.77)  1.542 (1.35-1.76)  1.483 (1.09-2.02) |  |  |
| Lee, 2015 [11] | Primary (ref) vs secondary  vs above high school | 1.31 (1.07-1.62)  2.54 (1.13-5.70) |  |  |
| Lee, 2010 [32] | None (ref) vs primary  vs secondary/high school  vs university | 1.51 (1.06-.16)  1.99 (1.36-2.92)  2.73 (1.71-4.35) |  |  |
| Leung, 2012 [12] | Secondary (ref)  vs none  vs primary | 0.20 (0.06-0.61)  0.31 (0.10-1.00) |  |  |
| Mukem, 2014 [36] | None (ref) vs primary  vs secondary  vs university  vs undergraduate | *BSE  1.62 (1.32-1.98)  2.37 (1.86-3.01)  2.75 (2.05-3.69)  2.82 (1.53-5.21) |  |  |
|  | None (ref) vs Primary vs university vs undergraduate | ** CBE 1.22 (1.00-1.50)  1.40 (1.04-1.90)  3.10 (1.59-6.02) |  |  |
|  | None (ref) vs university  vs undergraduate | ***Mammography  2.09 (1.12-3.89)  13.11 (5.69-30.2) |  |  |
| Okui, 2021 [24] | High school (ref)  vs secondary  vs primary | 0.71 (0.62-0.80)  0.49 (0.39-0.62) |  |  |
| Samah, 2012 [23] | None (ref) vs others | 8.50 (3.30-22.70) |  |  |
| Teo, 2013 [28] | Low educated (ref)  vs highly educated | *Ever had  1.74 (1.14-2.65)  **Regular  6.78 (3.54-12.99) |  |  |
| Tsunematsu, 2013 [25] |  |  | No (ref) vs yes | 1.67 (1.20-2.32) |
| Wang, 2013 [14] | Low educated (ref)  vs highly educated | 1.2 (1.0-1.6) |  |  |
| Yerramilli, 2015 [27] | Primary (ref)  vs secondary  vs high school | 2.57 (1.51-4.39)  3.71 (2.06-6.69) |  |  |
| You, 2019 [15] | Below high school (ref)  vs others | 1.30 (1.11-1.53) |  |  |
