# Supplementary material for: Socioeconomic factors affecting breast and cervical cancer screening compliance in Asian National Cancer Centers Alliance countries: a systematic review
Source: Epidemiol Health. 2025 Aug 28;47:e2025050. doi: 10.4178/epih.e2025050 (PMC12869128; doi:10.4178/epih.e2025050)
Supplement: Supplementary Material 4. — Factors associated with participation in cervical cancer screening (education level, household income) [file epih-47-e2025050-Supplementary-4.docx]

**Supplementary Material 4. Factors associated with participation in cervical cancer screening (education level, household income)**

|  | Education level | | Household income | |
| --- | --- | --- | --- | --- |
| First author, publish year | Group | OR (95% CI) | Group | OR (95% CI) |
| Ahmadipour, 2016 [19] | Below high school graduate (ref) vs others | *pap smear  2.50 (1.30-4.80) | < 1 million RLs (ref) vs above | *pap smear  3.00 (1.50-6.00) |
| Amin, 2020 [44] | None (ref) vs primary  vs high school  vs university | 1.76 (1.53-2.03)  2.47 (2.09-2.93)  2.24 (1.80-2.79) |  |  |
| Aminisani, 2016 [45] | Above secondary (ref)  vs none | 0.41 (0.23-0.73) |  |  |
| Anwar, 2018 [18] | Others (ref) vs above high school graduate | 1.58 (1.04-2.41) | Others (ref)  vs high income | 1.94 (1.40-2.69) |
| Chang, 2017 [52] | Primary(ref)  vs high school  vs university | 4.06 (1.46-11.29)  5.15 (1.86-14.32) | 1Q(ref) vs 2Q  vs 3Q  vs 4Q | 1.80 (1.12-2.91)  2.01 (1.23-3.28)  1.93 (1.19-3.14) |
| Chang, 2018 [53] |  |  | 1Q(ref) vs 2Q  vs 3Q | 1.12 (1.01-1.24)  1.25 (1.12-4.40) |
| Gu, 2010 [38] | Below secondary (ref)  vs high school | 3.00 (1.50-5.80) |  |  |
| Kulkarni, 2022 [43] | None (ref) vs primary  vs secondary  vs high school | 1.25 (1.11-1.42)  1.32 (1.22-1.44)  1.40 (1.18-1.67) |  |  |
| Lee, 2015 [11] | Primary (ref)  vs above high school | 2.54 (1.34-4.80) | 1Q (ref) vs 4Q  vs 5Q | 2.16 (1.08-4.31)  3.39 (1.66-6.92) |
| Lee, 2010 [31] |  |  | Lowest(ref)  vs 2-4^th^  vs highest | 1.31 (1.03-1.7)  1.63 (1.2-2.18) |
| Lee, 2013 [54] | Primary (ref)  vs secondary-high school  vs university | 1.71 (1.24-2.35)  1.73 (1.12-2.66) | Q1(ref) vs Q4 | 1.45 (1.08-1.94) |
| Lin, 2021 [39] | Below secondary (ref)  vs high school  vs university | 1.48 (1.29-1.69)  1.45 (1.22-1.71) | <2,000 RMB(ref)  vs 4,000-5,999  vs ≥6,000 | 1.37 (1.14-1.65)  1.47 (1.23-1.77) |
| Lin, 2021 [40] | Below secondary (ref)  vs high school  vs university | 1.57 (1.41-1.75)  2.19 (1.93-2.49) | <3,000 RMB(ref)  vs 5,000-9,999  vs ≥10,000 | 1.38 (1.23-1.56)  1.49 (1.26-1.77) |
| Liu, 2017 [41] | None (ref) vs primary  vs above secondary | 3.31 (1.85-5.93)  4.82 (2.72-8.56) |  |  |
| Mosayebi, 2018 [46]^)^ | Primary (ref)  vs secondary | 2.50 (1.30-4.80) |  |  |
| Siraj, 2019 [49] | Below secondary (ref)  vs high school  vs university | 2.50 (1.30-4.80)  3.00 (1.20-7.00) |  |  |
| Wee, 2012 [29] | Primary(ref)  vs secondary | 1.74 (1.04-2.93) |  |  |
| Wonwatcharanukul, 2014 [57] | None(ref) vs educated | 1.56 (1.02-2.38) |  |  |
| Yerramilli, 2015 [27] | Primary(ref)  vs secondary  vs high school | 3.17 (1.52-6.59)  4.16 (1.93-8.99) |  |  |
| You, 2019 [15] | High school or below(ref)  vs others | 1.21 (1.03-1.43) | ≤16,000 RMB (ref) vs above | 1.47 (1.32-1.65) |
| Zhang, 2023 [42] |  |  | <6,000 RMB (ref)  vs 10,001-20,000  vs >20,000 | 1.31 (1.07-1.59)  1.27 (1.01-1.61) |
