# Supplementary material for: Socioeconomic factors affecting breast and cervical cancer screening compliance in Asian National Cancer Centers Alliance countries: a systematic review
Source: Epidemiol Health. 2025 Aug 28;47:e2025050. doi: 10.4178/epih.e2025050 (PMC12869128; doi:10.4178/epih.e2025050)
Supplement: Supplementary Material 5. — Factors associated with participation in cervical cancer screening (marriage status, medical insurance) [file epih-47-e2025050-Supplementary-5.docx]

**Supplementary Material 5. Factors associated with participation in cervical cancer screening (marriage status, medical insurance)**

|  | Marriage status | | Medical insurance | |
| --- | --- | --- | --- | --- |
| First author, publish year | **Group** | **OR (95% CI)** | **Group** | **OR (95% CI)** |
| Al-Oseely, 2023 [48] |  |  | No (ref) vs yes | 2.22 (1.15-4.30) |
| Amin, 2020 [44] | Single (ref) vs married | 45.84 (29.46-71.34) |  |  |
| Aminisani, 2016 [45] |  |  | No (ref) vs yes | 2.31 (1.50-3.56) |
| Anwar, 2018 [18] |  |  | No (ref) vs yes | 1.57 (1.12-2.22) |
| Baussano, 2014 [37] | Single (ref) vs married | 5.23 (1.47-19.03) |  |  |
| Cui, 2022 [47] | Single (ref) vs married | 3.33 (2.35-4.74) |  |  |
| Gu, 2010 [38] | Married (ref) vs others | 0.11 (0.02-0.56) |  |  |
| Lin, 2021 [40] | Others (ref) vs married | 1.77 (1.49-2.11) | No (ref) vs yes | 1.58 (1.44-1.74) |
| Shin, 2022 [55] | Single (ref) vs married | 2.80 (2.29-3.44) |  |  |
| Sun, 2022 [13] | Single (ref) vs married | 1.51 (1.04-2.18) |  |  |
| Visanuyothin, 2015 [56] | Others (ref) vs married | 1.67 (1.10-2.50) |  |  |
| Zhang, 2023 [42] | Single (ref) vs married | 3.08 (2.54-3.73) |  |  |
