# Supplementary material for: Socioeconomic factors affecting breast and cervical cancer screening compliance in Asian National Cancer Centers Alliance countries: a systematic review
Source: Epidemiol Health. 2025 Aug 28;47:e2025050. doi: 10.4178/epih.e2025050 (PMC12869128; doi:10.4178/epih.e2025050)
Supplement: Supplementary Material 6. — Socioeconomic factors associated with participation in breast cancer screening in HDI 1 group (Education level & Household income) [file epih-47-e2025050-Supplementary-6.docx]

**Supplementary Material 6. Socioeconomic factors associated with participation in breast cancer screening in HDI 1 group (Education level & Household income)**

|  | Education level | | Household income | |
| --- | --- | --- | --- | --- |
| First Author (year), Country | Group | OR (95% CI) | Group | OR (95% CI) |
| Hahm(2010) [30] Korea |  |  | <$1000 vs ≥$5,000 | 1.73 |
| Lee(2010) [32] Korea | None (ref) vs primary vs secondary/high school vs university | 1.51(1.06-2.16) 1.99(1.36-2.92) 2.73(1.71-4.35) |  |  |
| Mukem(2014) [36]  Thailand | None (ref) vs Primary vs secondary vs university vs undergraduate | * BSE 1.62 (1.32-1.98) 2.37 (1.86-3.01) 2.75 (2.05-3.69) 2.82 (1.53-5.21) | Q1(ref) vs Q2 vs Q3 vs Q4 vs Q5 | * BSE 1.37 (1.17-1.60)  1.34 (1.15-1.56)  1.58 (1.34-1.85)  1.75 (1.45-2.10) |
|  | None (ref) vs Primary vs university vs undergraduate | ** CBE 1.22 (1.00-1.50)  1.40 (1.04-1.90)  3.10 (1.59-6.02) | Q1(ref) vs Q2 vs Q3 vs Q4 vs Q5 | ** CBE 1.24 (1.05-1.46) 1.21 (1.02-1.42) 1.18 (1.00-1.40) 1.59 (1.29-1.95) |
|  | None (ref) vs university vs undergraduate | *** Mammogram 2.09 (1.12-3.89) 13.11 (5.69-30.2) | Q1(ref) vs Q3 vs Q4 vs Q5 | *** Mammogram 1.61 (1.00-2.59) 2.13 (1.38-3.31) 3.23 (2.06-5.06) |
| Okui(2021) [24] Japan | high school (ref)  vs secondary vs primary | 0.71 (0.62-0.80) 0.49 (0.39-0.62) | Q4 (ref) vs Q3 vs Q2 vs Q1 | 0.84 (0.73, 0.98) 0.69 (0.59, 0.81) 0.64 (0.54, 0.77) |
| Son(2017) [35]  Korea |  |  | Lower income | 0.64 (0.42-0.98) |
| Teo(2013) [28] Singapore | Low educated (ref)  vs highly educated | *Ever had 1.74(1.14-2.65)  **Recommended 6.78(3.54-12.99) | < $4000(ref) vs ≥ $4000 | *Ever had 2.79(1.50-5.20)  **Recommended 3.75(2.00-7.00) |
