# Supplementary material for: Socioeconomic factors affecting breast and cervical cancer screening compliance in Asian National Cancer Centers Alliance countries: a systematic review
Source: Epidemiol Health. 2025 Aug 28;47:e2025050. doi: 10.4178/epih.e2025050 (PMC12869128; doi:10.4178/epih.e2025050)
Supplement: Supplementary Material 7. — Socioeconomic factors associated with participation in breast cancer screening in HDI 2~3 group (Education level & Employment & Family history) [file epih-47-e2025050-Supplementary-7.docx]

**Supplementary Material 7. Socioeconomic factors associated with participation in breast cancer screening in HDI 2~3 group (Education level & Employment & Family history)**

|  | Education level | | Being employed | | Family history | |
| --- | --- | --- | --- | --- | --- | --- |
| First Author (year), Country | Group | OR (95% CI) | Group | OR (95% CI) | Group | OR (95% CI) |
| Ahmadipour (2016) [19] Iran | Under high school (ref) vs higher High school | ***mammography 7.23(1.22-42.98) |  |  |  |  |
| Allahverdipour (2011) [20] Iran |  |  |  |  | No (ref) vs yes | 5.58 (1.27-24.52) |
| Anwar(2018) [18] Indonesia | Under high school (ref) vs higher High school | 4.26 (3.39-5.36) |  |  |  |  |
| Frie(2013) [16] India | None(ref) vs primary vs secondary  vs high school vs university or higher | *BSE 1.76 (1.47-2.10) 2.62 (2.17-3.17) 4.62 (3.53-6.06) 7.86 (5.47-11.30) | House wife (ref) vs Manual vs Others | *BSE 0.65 (0.48-0.7) 2.20 (1.83-2.65) | No (ref) vs yes | *BSE  1.24 (1.10-1.39)  **CBE 1.42 (1.20-1.69) |
| Gang(2013) [10] China | Highly educated (ref) vs low educated | 0.40 (0.211-0.765) |  |  |  |  |
| Ghanbari (2020) [22] Iran |  |  | House wife (ref) vs Employed | *BSE 1.65 (1.19-2.29) | No (ref) vs yes | ***mammography  1.60 (1.19-2.19) |
| Kulkarni (2019) [10]  India | None(ref) vs primary vs secondary vs high school | 1.413 (1.128-1.771) 1.542 (1.349-1.762) 1.483 (1.092-2.015) | Housewife/Student/retired (ref) vs employed | 0.75 (0.57-0.98) |  |  |
| Lee(2015) [11] China | Primary (ref) vs secondary vs high school | 1.31 (1.07-1.62) 2.54 (1.13-5.70) |  |  |  |  |
| Samah(2012) [23] Iran | None (ref) vs others | 8.50(3.30-22.70) | Housewife (ref) vs employed | 1.67 (1.20-2.32) |  |  |
| Sun(2022) [13] China |  |  | Unemployed (ref) vs employed | 1.64(1.30–2.05) |  |  |
| Wang(2013) [14]  China | Low educated (ref)  vs highly educated | 1.2 (95% CI 1.0–1.6) | Retired (ref) vs employed | 1.60 (1.20–2.10) |  |  |
