# Supplementary material for: Socioeconomic factors affecting breast and cervical cancer screening compliance in Asian National Cancer Centers Alliance countries: a systematic review
Source: Epidemiol Health. 2025 Aug 28;47:e2025050. doi: 10.4178/epih.e2025050 (PMC12869128; doi:10.4178/epih.e2025050)
Supplement: Supplementary Material 8. — Socioeconomic factors associated with participation in cervical cancer screening in HDI 1 group (Education level) [file epih-47-e2025050-Supplementary-8.docx]

**Supplementary Material 8. Socioeconomic factors associated with participation in cervical cancer screening in HDI 1 group (Education level)**

|  | Education level | |
| --- | --- | --- |
| First Author(year), Country | Group | OR (95% CI) |
| Chang(2017) [52]  Korea | Primary (ref) vs high school  vs university | 4.06 (1.46-11.29) 5.15 (1.86-14.32) |
| Lee(2013) [54]  Korea | Primary (ref) vs secondary-high school vs university | 1.71 (1.24-2.35) 1.73 (1.12-2.66) |
| Siraj(2019) [49] Malaysia | Below secondary (ref) vs high school  vs university | 2.50 (1.30-4.80) 3.00 (1.20-7.00) |
| Wee(2012) [29] Singapore | Primary (ref) vs secondary | 1.74 (1.04–2.93) |
| Wongwatcharanukulet(2014) [57]  Thailand | None (ref) vs educated | 1.56 (1.02-2.38) |
