# Supplementary material for: Socioeconomic factors affecting breast and cervical cancer screening compliance in Asian National Cancer Centers Alliance countries: a systematic review
Source: Epidemiol Health. 2025 Aug 28;47:e2025050. doi: 10.4178/epih.e2025050 (PMC12869128; doi:10.4178/epih.e2025050)
Supplement: Supplementary Material 9. — Socioeconomic factors associated with participation in cervical cancer screening in HDI 2~3 group (Education level & Household income) [file epih-47-e2025050-Supplementary-9.docx]

**Supplementary Material 9. Socioeconomic factors associated with participation in cervical cancer screening in HDI 2~3 group (Education level & Household income)**

|  | Education level | | Household income | |
| --- | --- | --- | --- | --- |
| First Author (year), Country | Group | OR (95% CI) | Group | OR (95% CI) |
| Ahmadipour (2016) [19]  Iran | below high school graduate(ref) vs others | *pap smear  2.50 (1.30-4.80) |  |  |
| Amin(2020) [44] Iran | None (ref) vs primary  vs high school  vs university | 1.76 (1.53-2.03) 2.47 (2.09-2.93)  2.24 (1.80-2.79) |  |  |
| Aminisani(2016) [45] Iran | Above secondary (ref) vs none | 0.41 (0.23-0.73) |  |  |
| Anwar(2018) [18] Indonesia | Others (ref) vs above high school graduate | 1.58 (1.04-2.41) | Others (ref) vs high income | 1.94 (1.40-2.69) |
| Gu(2010) [38] China | Below secondary (ref)  vs high school | 3.00 (1.50-5.80) |  |  |
| Kulkarni(2022) [43] India | None (ref) vs primary  vs secondary  vs high school | 1.25 (1.11-1.42) 1.32 (1.22-1.44) 1.40 (1.18-1.67) |  |  |
| Lee(2015) [11] China | Primary (ref) vs above high school | 2.54 (1.34-4.80) | 1Q vs 4Q  vs 5Q | 2.16 (1.08-4.31) 3.39 (1.66-6.92) |
| Lin(2021) [39] China | Below secondary (ref)  vs high school  vs university | 1.48(1.29-1.69) 1.45(1.22-1.71) | <2,000 RMB (ref)  vs 4,000-5,999  vs ≥6,000 | 1.37 (1.14-1.65) 1.47 (1.23-1.77) |
| Lin(2021) [40] China | Below secondary (ref)  vs high school  vs university | 1.57 (1.41-1.75) 2.19 (1.93-2.49) | <3,000 RMB (ref)  vs 5,000-9,999  vs ≥10,000 | 1.38 (1.23-1.56) 1.49 (1.26-1.77) |
| Liu(2017) [41] China | None (ref)  vs primary  vs above secondary | 3.31(1.85–5.93) 4.82(2.72–8.56) |  |  |
| Mosayebi(2018) [46] Iran | Primary (ref) vs secondary | 2.50 (1.30-4.80) |  |  |
| Yerramilli(2015) [27] Mongolia | Primary (ref) vs secondary  vs high school | 3.17 (1.52–6.59) 4.16 (1.92–8.99) |  |  |
| You(2019) [15] China | High school or below (ref) vs others | 1.21 (1.03-1.43) | ≤16,000 RMB (ref)  vs above | 1.47 (1.32-1.65) |
| Zhang (2023) [42] China |  |  | <6,000 RMB (ref)  vs 10,001-20,000  vs > 20,000 | 1.31 (1.07–1.59) 1.27 (1.01–1.61) |
