# Supplementary material for: Socioeconomic factors affecting breast and cervical cancer screening compliance in Asian National Cancer Centers Alliance countries: a systematic review
Source: Epidemiol Health. 2025 Aug 28;47:e2025050. doi: 10.4178/epih.e2025050 (PMC12869128; doi:10.4178/epih.e2025050)
Supplement: Supplementary Material 10. — Socioeconomic factors associated with participation in cervical cancer screening in HDI 2~3 group (Marriage status & Employment) [file epih-47-e2025050-Supplementary-10.docx]

**Supplementary Material 10. Socioeconomic factors associated with participation in cervical cancer screening in HDI 2~3 group (Marriage status & Employment)**

|  | Marriage status | | Being employed | |
| --- | --- | --- | --- | --- |
| First Author(year), Country | Group | OR (95% CI) | Group | OR (95% CI) |
| Ahmadipour (2016) [19] Iran |  |  | Housewife (ref)  vs employed | *pap smear  2.50(1.20-5.30) |
| Amin(2020) [44] Iran | Single (ref) vs married | 45.84 (29.46-71.34) | Unemployed (ref)  vs employed | 0.83 (0.71-0.99) |
| Baussano (2014) [37] Bhutan | Single (ref) vs married | 5.23 (1.47-19.03) | Housewife (ref) vs Manual | 2.10 (1.22-3.62) |
| Gu(2010) [38] China | Married (ref) vs others | 0.11 (0.02-0.56) |  |  |
| Lin(2021) [40] China | Others (ref) vs married | 1.77 (1.49-2.11) | Unemployed (ref)  vs employed | 1.19 (1.06-1.35) |
| Sun(2022) [13] China | Single (ref) vs married | 1.51 (1.04–2.18) | Unemployed (ref)  vs employed | 1.64 (1.30–2.05) |
| Yerramilli(2015) [27] Mongolia |  |  | Housewife/retired (ref) vs employed | 1.53 (1.09–2.14) |
| Zhang(2023) [42] China | Single (ref) vs married | 3.08 (2.54–3.73) | Medical worker (ref)  vs student vs manual vs unemployed | 0.28 (0.17–0.48) 0.62 (0.42–0.94) 0.50 (0.34–0.76) |
