# Supplementary material for: Socioeconomic factors affecting breast and cervical cancer screening compliance in Asian National Cancer Centers Alliance countries: a systematic review
Source: Epidemiol Health. 2025 Aug 28;47:e2025050. doi: 10.4178/epih.e2025050 (PMC12869128; doi:10.4178/epih.e2025050)
Supplement: Supplementary Material 11. — Socioeconomic factors associated with participation in breast cancer screening in countries with National Screening Program (Household income) [file epih-47-e2025050-Supplementary-11.docx]

**Supplementary Material 11. Socioeconomic factors associated with participation in breast cancer screening in countries with National Screening Program (Household income)**

|  | Household income | |
| --- | --- | --- |
| First Author (year), Country | Group | OR (95% CI) |
| Chan(2021) [28] Singapore | < $2,000 (ref) vs ≥ $10,000 | *Ever had 1.20 (1.11-1.30)  **Recommended 1.17 (1.37-2.13) |
| Hahm(2010) [30] Korea | < $1000 (ref) vs ≥ $5,000 | 1.73 (p<0.01) |
| Okui(2021) [24] Japan | 4Q (ref) vs 3Q vs 2Q vs 1Q | 0.84 (0.73-0.98) 0.69 (0.59-0.81) 0.64 (0.54-0.77) |
| Son(2017) [35] Korea | Lower income | 0.64 (0.42-0.98) |
| Teo(2013) [28] Singapore | <$4000 (ref) vs ≥$4000 | *Ever had  2.79 (1.50-5.20) **Recommended  3.75 (2.00-7.00) |
| Wee (2012) [29] Singapore | ≤$500 (ref) vs > $500, <$1500 | 1.58 (1.20–2.08) |
