# Supplementary material for: Socioeconomic factors affecting breast and cervical cancer screening compliance in Asian National Cancer Centers Alliance countries: a systematic review
Source: Epidemiol Health. 2025 Aug 28;47:e2025050. doi: 10.4178/epih.e2025050 (PMC12869128; doi:10.4178/epih.e2025050)
Supplement: Supplementary Material 12. — Socioeconomic factors associated with participation in breast cancer screening in countries without National Screening Program (Education level & Employment & Region) [file epih-47-e2025050-Supplementary-12.docx]

**Supplementary Material 12. Socioeconomic factors associated with participation in breast cancer screening in countries without National Screening Program (Education level & Employment & Region)**

|  | Education level | | Being employed | | Living urban | |
| --- | --- | --- | --- | --- | --- | --- |
| First Author (year), Country | Group | OR (95% CI) | Group | OR (95% CI) | Group | OR (95% CI) |
| Anwar(2018) [18] Indonesia | Below high school graduate (ref) vs others | 4.26 (3.39-5.36) |  |  |  |  |
| Frie(2013) [16] India | None (ref) vs primary  vs secondary  vs high school  vs above university | 1.76 (1.47-2.10) 2.62 (2.17-3.17) 4.62 (3.53-6.06) 7.86 (5.47-11.30) | House wife (ref) vs Manual House wife (ref) vs Others | 0.65 (0.48-0.7) 2.20 (1.83-2.65) |  |  |
| Gang(2013) [10] China | Highly educated (ref) vs low educated | 0.40 (0.21-0.77) |  |  |  |  |
| Kulkarni(2019) [17] India | None (ref) vs primary  vs secondary  vs high school | 1.41 (1.13-1.77) 1.54 (1.35-1.76) 1.48 (1.09-2.02) | Housewife/Student/retired (ref) vs employed | 0.75 (0.57-0.98) |  |  |
| Lee(2015) [11] China | Primary (ref) vs secondary  vs above high school | 1.31 (1.07-1.62) 2.54 (1.13-5.70) |  |  | Rural (ref) vs urban | 2.31 (1.61-3.31) |
| Leung(2012) [12] China | Secondary (ref)  vs none  vs primary | 0.20 (0.06–0.61) 0.31 (0.10–1.00) |  |  |  |  |
| Mukem(2014) [36] Thailand | None (ref) vs primary  vs secondary  vs university  none(ref) vs undergraduate | *BSE 1.62 (1.32-1.98) 2.37 (1.86-3.01) 2.75 (2.05-3.69) 2.82 (1.53-5.21) |  |  |  |  |
|  | None (ref) vs primary  vs university  vs undergraduate | **CBE 1.22 (1.00-1.50)  1.40 (1.04-1.90)  3.10 (1.59-6.02) |  |  |  |  |
|  | None (ref) vs university  vs undergraduate | ***Mammogram 2.09 (1.12-3.89) 13.11 (5.69-30.2) |  |  | Rural (ref) vs urban | 1.97 (1.54-2.51) |
| Sun(2022) [13] China |  |  | Unemployed (ref)  vs employed | 1.64 (1.30–2.05) |  |  |
| Wang(2013) [14] China | Low educated (ref)  vs highly educated | 1.20 (1.00–1.60) | Retired (ref) vs employed | 1.60 (1.20–2.10) |  |  |
| You(2019) [15] China | Below high school (ref)  vs others | 1.30 (1.11-1.53) |  |  |  |  |
